# Leclercia adecarboxylata: An Emerging Pathogen Among Pediatric Infections

**DOI:** 10.7759/cureus.8049

**Published:** 2020-05-10

**Authors:** Jonathan Keyes, Evan P Johnson, Monica Epelman, Adriana Cadilla, Syed Ali

**Affiliations:** 1 Internal Medicine, University of Central Florida College of Medicine, Orlando, USA; 2 Orthopaedics, University of Central Florida College of Medicine, Orlando, USA; 3 Radiology, Nemours Children's Hospital/University of Central Florida College of Medicine, Orlando, USA; 4 Infectious Disease, Nemours Children's Hospital, Orlando, USA; 5 Inpatient Pediatrics, Nemours Children's Hospital, Orlando, USA

**Keywords:** leclercia adecarboxylata, pediatrics, infections, bacteria, cellulitis, urinary tract infection, emerging, chronic kidney disease

## Abstract

*Leclercia adecarboxylata *is a gram-negative bacillus of the Enterobacteriaceae family. It is a rare human pathogen that is often acquired via wound and/or contact with aquatic environment. Although multiple cases of *L. adecarboxylata *infections are described in the adult population, few have been documented in pediatrics. We will present two cases of *L. adecarboxylata *infections in the pediatric population. The first is a case of cellulitis in an 11-year-old male patient after a penetrating wound. The second is a first-documented urinary tract infection in a 16-year-old male patient with chronic kidney disease. Both patients were successfully treated with antibiotics and surgical intervention, if necessary. These cases highlight the growing emergence of this bacterium in the pediatric population and the need to become more aware of its threat even in patients who are immunocompetent.

## Introduction

*Leclercia adecarboxylata *is a mobile gram-negative bacillus that is generally sensitive to most antibiotics and was first described by Leclerc in 1962 as *Escherichia adecarboxylata*, but was reclassified as *L. adecarboxylata *after further studies showed that it belonged to a different genus [1,2]. It is an extremely rare human pathogen that commonly infects immunocompromised individuals, with few cases documented in adults and even fewer in pediatrics. Of the documented pediatric cases, *L. adecarboxylata *is known to cause cellulitis from wound infections and/or contact with contaminated water, consistent with multiple known cases in the adult counterpart [3-7]. However, it has also been documented in pediatrics to cause folliculitis, peritonitis from peritoneal dialysis, and bacteremia in preterm infants and a patient with acute lymphoblastic leukemia [8-12]. Nonetheless, compared to adults, there are fewer cases of *L. adecarboxylata *documented in pediatrics. We present two cases to highlight the need for growing awareness of* L. adecarboxylata* as it is becoming an emerging infection in the pediatric population. The first is a classic case of cellulitis after a penetrating foot infection in an immunocompetent boy and the second is a first-documented urinary tract infection (UTI) in a patient with chronic kidney disease (CKD).

## Case presentation

**Figure 1:**
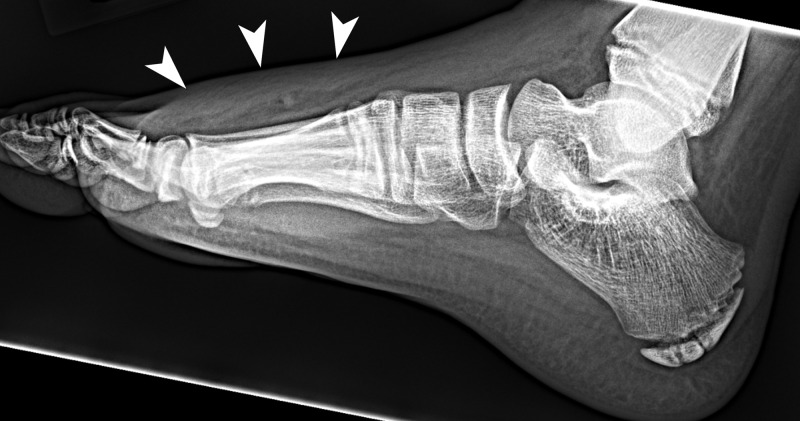
Lateral radiograph of the right foot Initial lateral radiograph of the right foot shows focal soft tissue swelling (arrowheads) overlying the dorsum of the foot but no radiopaque foreign body.

The first case is an 11-year-old boy with no past medical history who fell off his bike, hitting a branch, and sustained a penetrating wound by a wood splinter on the dorsal right foot. The mother cleaned the wound with water. The swelling persisted without drainage overnight and the next day, he went to the emergency department (ED) and was discharged on oral cephalexin to prevent cellulitis. After two days of antibiotic, the swelling worsened with draining yellow pus. Throughout this time period, the mother has continued to wash the wound with water and has noticed pieces of wood coming out. The patient was hospitalized after failing outpatient treatment. He was afebrile at the time of admission. Initial lateral radiograph of the right foot (Figure 1) on admission showed focal soft tissue swelling overlying the dorsum of the foot, but no radiopaque foreign body was identified at the time. The patient was empirically started on intravenous (IV) clindamycin. Pus specimen grew* L. adecarboxylata* that was resistant to ampicillin but sensitive to ampicillin/sulbactam, cefazolin, gentamicin, and tobramycin. Because the patient was experiencing optimal clinical response to IV clindamycin, it was decided by infectious disease to continue IV clindamycin with the eventual transition to oral clindamycin upon discharge. However, two weeks after discharge, the wound was draining again.

**Figure 2:**
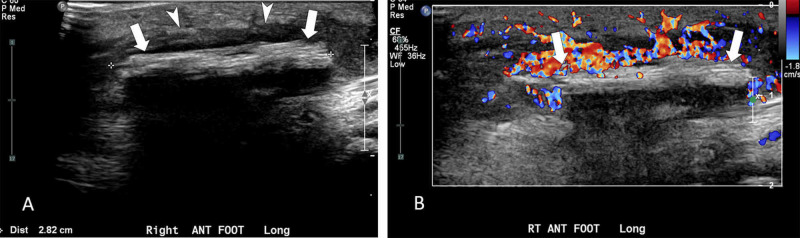
Longitudinal greyscale and color Doppler of the right foot Longitudinal grayscale (A) and color Doppler (B) ultrasound images obtained on re-admission show an echogenic structure (arrows), believed to represent a foreign body measuring approximately 2.8 cm in length and located approximately 10 mm deep to the skin surface. Hypoechoic granulation tissue (arrowheads in A) is seen adjacent to the foreign body, which is hypervascular on color Doppler interrogation (B).

An ultrasound (Figure 2) at the ED showed a 0.5 x 0.8 x 2.8 cm echogenic structure, suspected to be a retained foreign body or granulation tissue. Due to the persistent nature of the wound, the patient was referred to orthopedics for a wound exploration and debridement. Granulation tissue was debrided. No obvious foreign body was encountered. Intraoperative culture was negative for organisms, and the patient was discharged home with oral cephalexin. He had no further complications at follow-up. 

**Figure 3:**
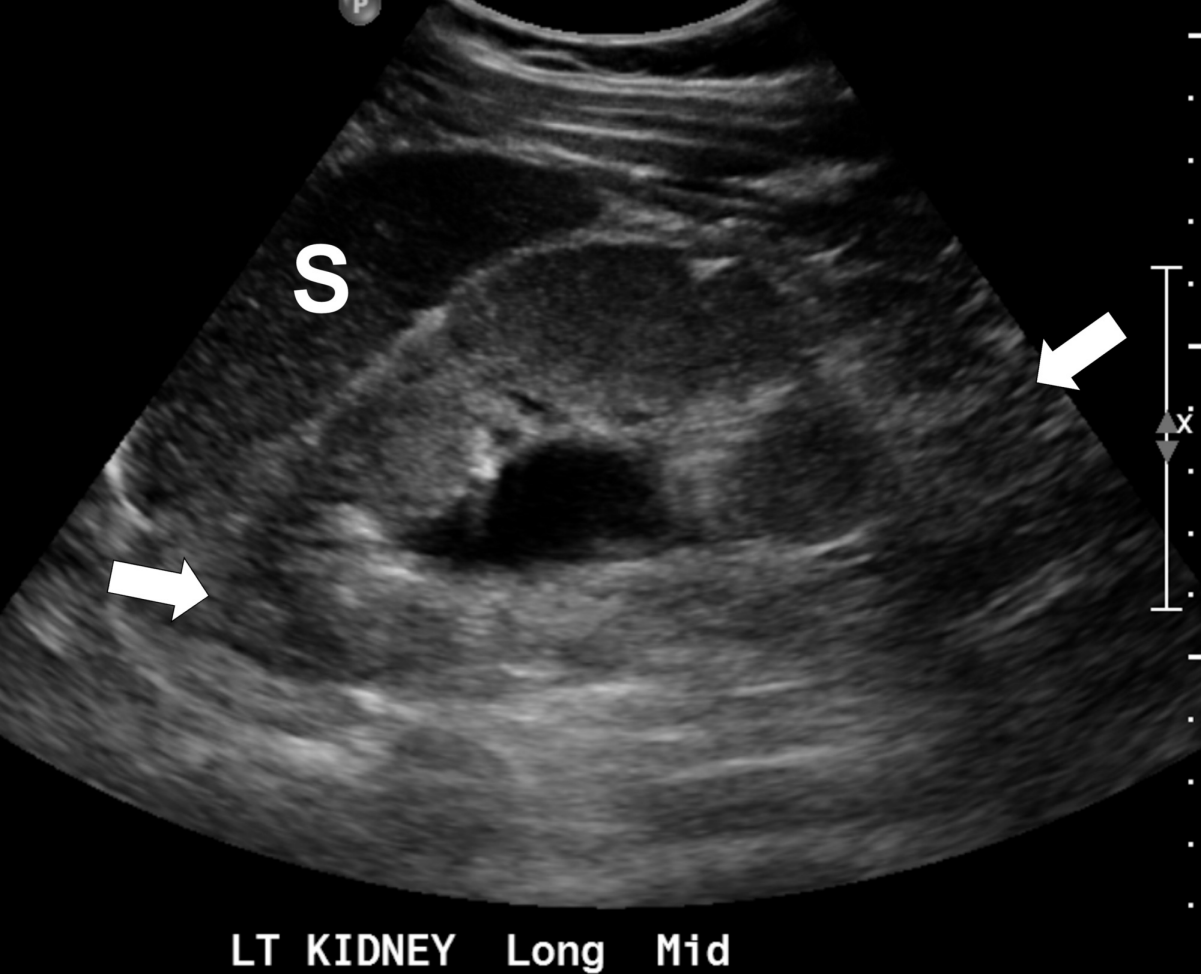
Longitudinal ultrasound of the left kidney Longitudinal ultrasound image of the left kidney (arrows) shows a top normal size kidney with mild pelviectasis, and mild, diffuse increase in parenchymal echogenicity with poor corticomedullary differentiation. The renal parenchyma appears mildly increased in echogenicity when compared to the adjacent splenic (S) parenchyma.

The second case is a 16-year-old male with a history of high-grade reflux nephropathy, solitary left kidney, and neurogenic bladder on intermittent Foley catheterization every three hours. The patient had stage 4 CKD with an elevated serum creatinine of 6.1 mg/dL (baseline 3.0 mg/dL) in December 2016, which warranted the placement of a left nephrostomy tube. The nephrostomy tube was removed in March 2017. He presented to the ED with nausea and vomiting four days after left nephrostomy tube removal. There was no trauma or contact with contaminated water. He was afebrile and denied flank pain. The nephrostomy site did not appear to be infected. Creatinine was 5.0 mg/dL and blood urea nitrogen (BUN) was 55 mg/dL. The mother was concerned for pyelonephritis, but ultrasound at the time did not show any significant changes in the left kidney. A midstream urine culture grew 70,000 cfu/mL *L. adecarboxylata*. Susceptibility panel showed that it was sensitive to ampicillin, ampicillin/sulbactam, cefazolin, gentamicin, nitrofurantoin, and tobramycin. The patient was discharged home with oral cephalexin. A week later, the patient presented back at the ED with left flank pain and intermittent vomiting, but no fever. He was not compliant with the antibiotic regimen due to complex social situation. He was hospitalized and started on IV ceftriaxone. Creatinine improved at 4.3 mg/dL, BUN remained elevated at 53 mg/dL, and albumin was decreased at 3.5 g/dL. Urinalysis was significant for 2+ proteinuria, but absence of white blood cells, leukocyte esterase, nitrite, and bacteria. Ultrasound (Figure 3) showed a hypodense mass in the left mid-kidney concerning for a possible abscess or a callous reaction to prior nephrostomy. Urine culture was negative for organisms, and the patient no longer experienced any left flank pain after 24 hours. He was afebrile and denied nausea and vomiting. The patient was discharged home on cefdinir. There was no symptomatic recurrence, and renal ultrasound at follow-up was stable.

## Discussion

*L. adecarboxylata* is a human pathogen that is infrequently documented in the adult population and even less so in pediatrics. Although *L. adecarboxylata* was first described in 1967, it only began to generate attention in pediatric infectious disease during the past 10 years [1]. A literature review of cases published in English on PubMed using the search term “Leclercia adecarboxylata” showed 44 cases of *L. adecarboxylata* adult infections and only nine pediatric infections. Of the documented pediatric infections, two were between 2000 and 2004, four between 2010 and 2014, and five between 2015 and 2019 (including the two cases reported here). Although* L. adecarboxylata* is still a relatively uncommon infection, there has been an increase in the number of reported pediatric cases throughout the recent years. Indeed, it has been speculated that the number of *L. adecarboxylata* infections could be under-reported given the high degree of phenotypic overlap between *L. adecarboxylata* and *Escherichia coli *and the inability for an automated diagnostic system to reliably distinguish between the two [6].

*L. adecarboxylata *was documented as an opportunistic pathogen that frequently causes monomicrobial infections in the immunocompromised individuals [13]. While this is true as most of the documented pediatric cases are of patients who were immunocompromised (Table 1), it is also worth noting that infections are gradually occurring in the immunocompetent patients such as our first patient presenting with cellulitis. Three other cases have been documented in pediatric patients with intact immune function: a nine-year-old girl with cellulitis after a left foot puncture wound, a two-year-old boy with cellulitis after a right thumb laceration, and a 12-year-old boy with folliculitis after exposure to aquatic environment [3,4,8]. Based on these case reports, the cause of infection in our patient either stemmed from retained foreign body secondary to penetrating wound or improper wound cleaning with contaminated water. The patient was exposed to both risk factors, and it is difficult to distinguish the underlying cause of his infection. Although our patient was suspected to have a retained foreign object on re-admission based on the ultrasound (Figure 2), no obvious foreign body was encountered during wound exploration. We suspect that the echogenic structure was likely to be granulation tissue and that healing had already taken place, especially since intraoperative culture did not show any organisms. Our first case is presented to raise awareness of *L. adecarboxylata *in pediatric infections and to demonstrate its emerging presence even among those who are immunocompetent.

**Table 1:** Features of Leclercia adecarboxylata pediatric infection case reports A review of all documented *L. adecarboxylata* infections published in English on PubMed for the pediatric population.

Immune Deficiency/Underlying Condition	Culture Source	Coinfection(s)	Reference
None	Wound	None	Grantham et al. [3]
None	Wound	None	Hurley et al. [4]
None	Folliculitis	None	Broderick et al. [8]
End-stage renal disease	Peritoneal fluid	None	Fattal and Deville [9]
Premature birth	Blood	None	Myers et al. [10]
Premature birth	Blood	None	Nelson et al. [11]
Acute lymphoblastic leukemia	Blood	Staphylococcus aureus	Longhurst and West [12]
Leukopenia and neutropenia	Blood	None	Sethi et al. [14]
Acute lymphoblastic leukemia	Cellulitis	None	Shah et al. [15]

To our knowledge, the second case is the first published* L. adecarboxylata* UTI in a pediatric patient. *L. adecarboxylata* infections have been reported in the adult population after foreign body placements such as a central line or a peritoneal catheter [16,17]. Similarly, a child was infected with *L. adecarboxylata* during peritoneal dialysis for end-stage renal disease [9]. Our patient had a chronic history of intermittent Foley catheterization due to neurogenic bladder, which could have increased his risk of UTI from* L. adecarboxylata*. At the time of his first admission, we did not find evidence of pyelonephritis given the unremarkable kidney ultrasound. On re-admission, pyelonephritis was suspected based on the significant change in renal ultrasound (Figure 3). This suspicion was further supported by his uncontrolled UTI resulted from medication non-compliance and possibly inadequate immune response secondary to CKD. We know from past reports that* L. adecarboxylata* can act as an opportunistic pathogen for the immunocompromised individuals, with one case where mortality was observed in an immunocompromised pediatric patient [13,14]. CKD has been known for its association with acquired immunosuppression. While the patient did not demonstrate neutropenia, his state of uremia can still contribute to an inadequate immune response given that uremia can inhibit immune cell activation [18]. The patient also demonstrated proteinuria on the second admission. Unfortunately, his immunoglobulin level was not trended between the two admissions. Nonetheless, we have a high clinical suspicion that the patient may have lost immunoglobulin through urinary excretion in the past. This may have also increased his risk to *L. adecarboxylata *infection. The case again highlights the need for growing awareness of *L. adecarboxylata* infections in the pediatric population. More importantly, given the increased frequency of intermittent catheterization and nephrostomy as a result of congenital kidney diseases, it is imperative to be cognizant of *L. adecarboxylata* in this more vulnerable patient population in order to obtain an early diagnosis for proper treatment and minimize mortality.

## Conclusions

We present two cases of* L. adecarboxylata* infections in the pediatric population, one who was immunologically intact with cellulitis and one who was immunologically weakened with UTI. Diagnosis was made based on laboratory findings. Proper antibiotic treatments and surgical intervention, if necessary, were provided with successful resolution of the infection in both cases. The cases highlight the emerging presence of *L. adecarboxylata* among pediatric infections and care providers should increase their awareness of this phenomenon. . 
